# Advanced multimodality MR imaging of a cerebral nocardiosis abscess in an immunocompetent patient with a focus on Amide Proton Transfer weighted imaging

**DOI:** 10.1259/bjrcr.20190122

**Published:** 2020-09-29

**Authors:** Elisabeth Sartoretti, Thomas Sartoretti, Annina Gutzwiller, Urs Karrer, Christoph Binkert, Arash Najafi, David Czell, Simon Beyeler, Sabine Sartoretti-Schefer

**Affiliations:** 1 Institut für Radiologie, Kantonsspital Winterthur, Brauerstrasse 15, 8401 Winterthur, Switzerland; 2 Klinik für Innere Medizin, Kantonsspital Winterthur, Brauerstrasse 15, 8401 Winterthur, Switzerland; 3 Klinik für Innere Medizin, Zuger Kantonsspital, Landhausstrasse 11, 6340 Baar, Switzerland

## Abstract

Cerebral nocardiosis abscess is a very rare entity in an immunocompetent patient. In this case report multiparametric and multimodality MR imaging characteristics of a pyogenic brain abscess caused by Nocardia Farcinica are discussed with a specific focus on amide proton transfer weighted imaging as a modern non-invasive, molecular MR imaging method which detects endogenous mobile protein and peptide concentration and tissue pH changes in pathologic brain lesions. The imaging characteristics are reviewed and discussed in respect to possible differential diagnoses, especially malignant tumorous lesions.

## Introduction

Nocardia is a Gram-positive bacillus, that causes cutaneous, ocular and central nervous system (CNS) infections especially in immunocompromised patients with deficiencies in the cell-mediated immunity.^1^ CNS infections (meningitis and rarely brain abscess) occur in 9–44% of these patients.^1,2^ However, in general, nocardial brain abscesses appear very rarely and only represent^1,2^ 1–2% of all brain abscesses.^3^

In our report, we describe a rare case of a brain abscess due to Nocardia Farcinica in an immunocompetent patient. The patient was referred to the emergency unit after an epileptic seizure. CT imaging showed a tumorous frontal mass lesion. In our department, all patients with brain tumors are examined with an advanced comprehensive multimodal brain MRI imaging protocol^4–6^ for further characterization of the tumorous lesion. This protocol consists of morphological MR sequences (*T*_2_ weighted (*T*_2_W) turbo spin echo (TSE), *T*_1_W pre-contrast turbo field echo (TFE), post-contrast *T*_1_W m-Dixon TFE, three-dimensional fluid attenuated inversion recovery (FLAIR), diffusion weighted imaging (DWI), susceptibility weighted imaging (SWI)) and of advanced imaging modalities (Diffusion tensor imaging (DTI), dynamic susceptibility contrast (DSC) perfusion-weighted imaging (PWI), dynamic contrast enhanced (DCE) PWI, single voxel spectroscopy and amide proton transfer weighted (APTw) imaging).

On morphological MRI (DWI, SWI, *T*_2_W and post-contrast *T*_1_W imaging) as well as on advanced MR imaging based on DSC PWI and DTI the frontal mass lesion could be diagnosed as a brain abscess although spectroscopy data and k-trans values resulting from DCE PWI were inconclusive. The diagnosis of brain abscess caused by Nocardia farcinica was finally confirmed following neurosurgical intervention.

While the imaging characteristics of Nocardia brain abscesses are well known for certain standard imaging modalities such as DWI or DSC, the imaging characteristics on APTw images have not been described to this date. With APTw imaging being a modern non-invasive, molecular MRI method that has shown promise in imaging tumors and infective mass lesions,^7^ a detailed description of these imaging characteristics may be of interest as APTw imaging may become a valuable future asset in imaging infective mass lesions especially in complex cases where the diagnosis can not be ascertained with currently available imaging modalities.

## Case report

A 76-year-old patient with chronic obstructive pulmonary disease, chronic atrial fibrillation and alcohol overconsumption was admitted to the hospital in a soporous to comatose state (Glasgow coma scale GCS 5) after persistent focal-complex epileptic seizures. On emergency CT imaging, a focal peripherally contrast enhancing mass lesion in the frontal lobe on the left side was detected. Advanced multimodal brain tumor imaging was performed for further characterization of the tumorous lesion.

**Figure 1.**
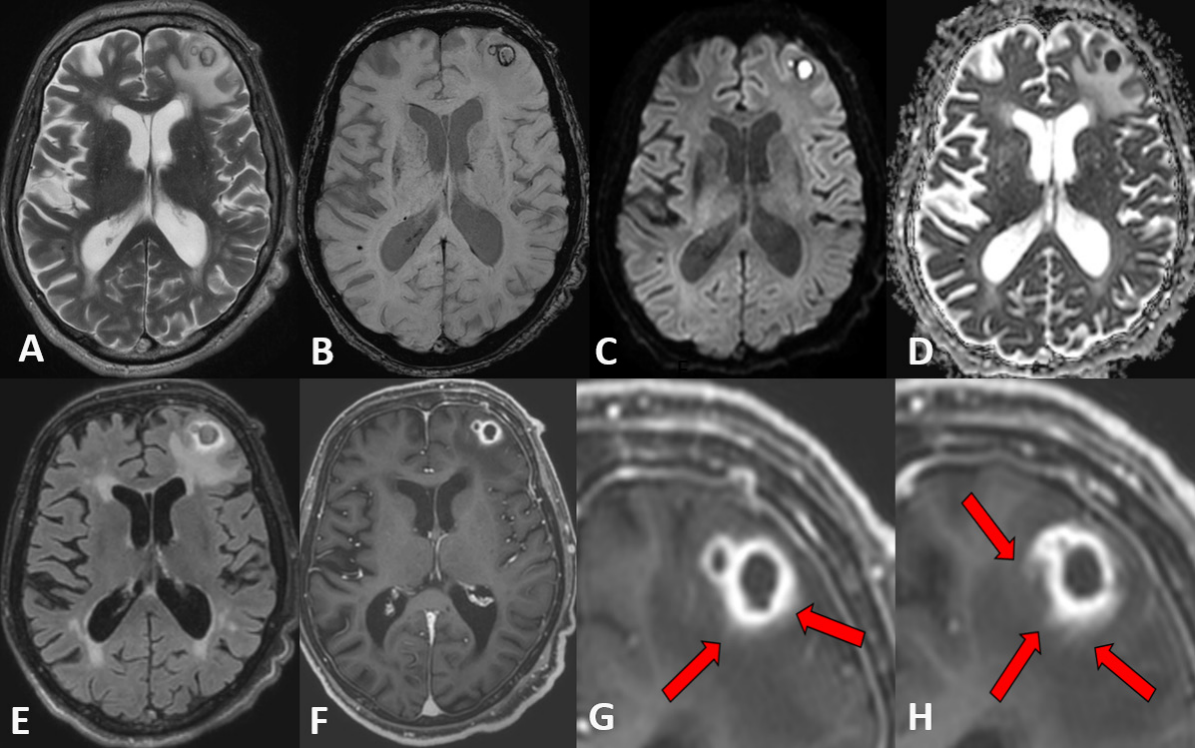
Transverse *T*_2_W TSE (A) , SWI (Figure 1B), DWI (Figure 1C), ADC map (Figure 1D) and FLAIR (Figure 1E) images and post-contrast *T*_1_W TFE (Figure 1F–1H) presenting the two peripherally contrast enhancing confluent lesions in the left frontal white matter Red arrows show indistinct contrast enhancement in the surrounding tissue. ADC, apparent diffusion co-efficient; DWI,diffusion-weighted imaging; FLAIR, fluid attenuated inversion recovery; SWI, susceptibility-weighted imaging; *T*_2_W, *T*_2_weighted; TFE, turbo field echo.

On morphological brain MRI (Figure 1), two confluent and centrally necrotic lesions (of 7 and 14 mm diameter) with peripheral irregular contrast enhancing rims (Figure 1F–H), hypointense on *T*_2_W TSE (Figure 1A) and with double rim sign on SWI (Figure 1B), and with substantial perifocal edema on FLAIR (Figure 1E) and *T*_2_W images (Figure 1A) were visible in the left middle frontal gyrus. On SWI (Figure 1B), the outer hypointense rims were irregular with central hypointense protrusions. The contrast enhancing rims (Figure 1F–H) were thick and irregular, hyperintense on FLAIR (Figure 1E), without well-delineated borders and with slight DCE in the surrounding tissue (red arrows in Figure 1G–H).

**Figure 2.**
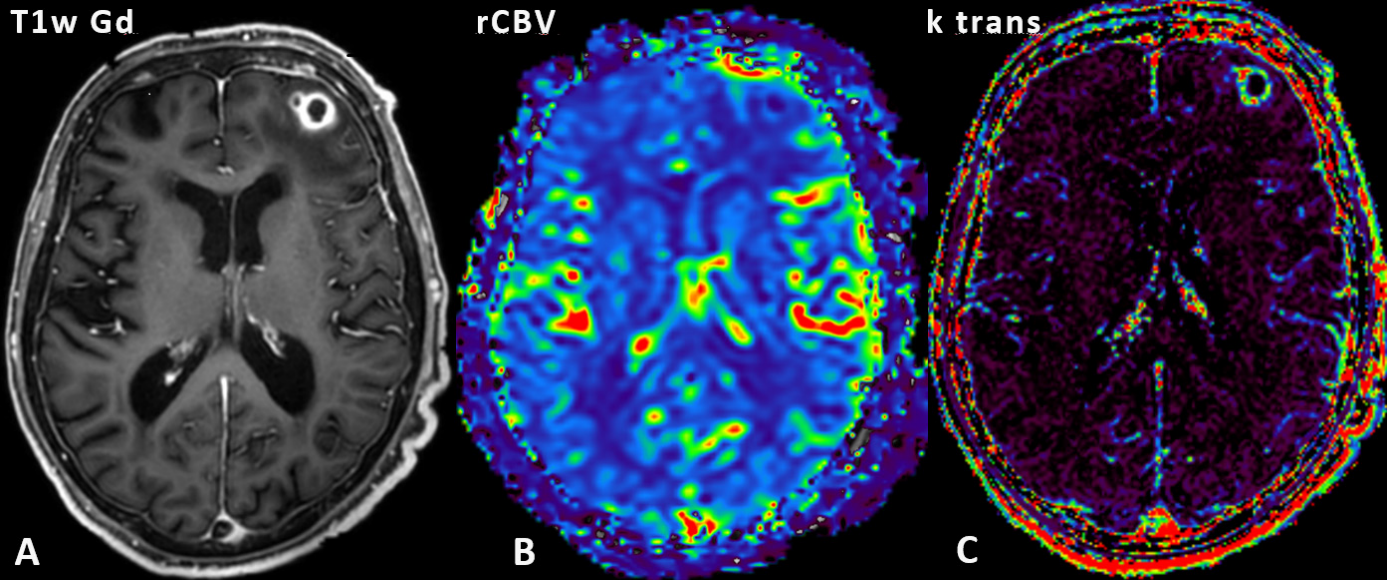
DSC perfusion with rCBV map: A *T*_1_W TFE m-Dixon transverse image (Figure 2A) is underlaid on the rCBV map (Figure 2B). The k-trans map is visible in Figure 2C. The contrast enhancing rim does not show any hyperperfusion. DSC, dynamic susceptibility contrast; *T*_1_W, *T*_1_ weighted; TFE, turbo field echo.

On DSC perfusion, the relative cerebral blood volume of the contrast enhancing rim compared to normal white matter in the centrum semiovale (rCBV) was not pathologically increased (Figure 2A and B) with a rCBV of 0.86. On DCE perfusion, the k-trans values of the contrast enhancing rim were evaluated (Figure 2C) and varied between 22 and 41 × 10^−3^/min compared to a value of 2.3 × 10^−3^/min of normal contralateral white matter. A high signal intensity increase on DCE perfusion lead to a maximum relative enhancement of 81% compared to 4.6% of normal white matter resulting in an ascending curve without wash-out.

The necrotic center presented with homogeneous hyperintensity on DWI (Figure 1C) and with severely reduced apparent diffusion coefficient (ADC) (Figure 1D) corresponding to diffusion restriction with a mean ADC value of 0.4 × 10^−3^ mm2/s and with a central fractional anisotropy (FA) value of 0.28 on DTI.

**Figure 3.**
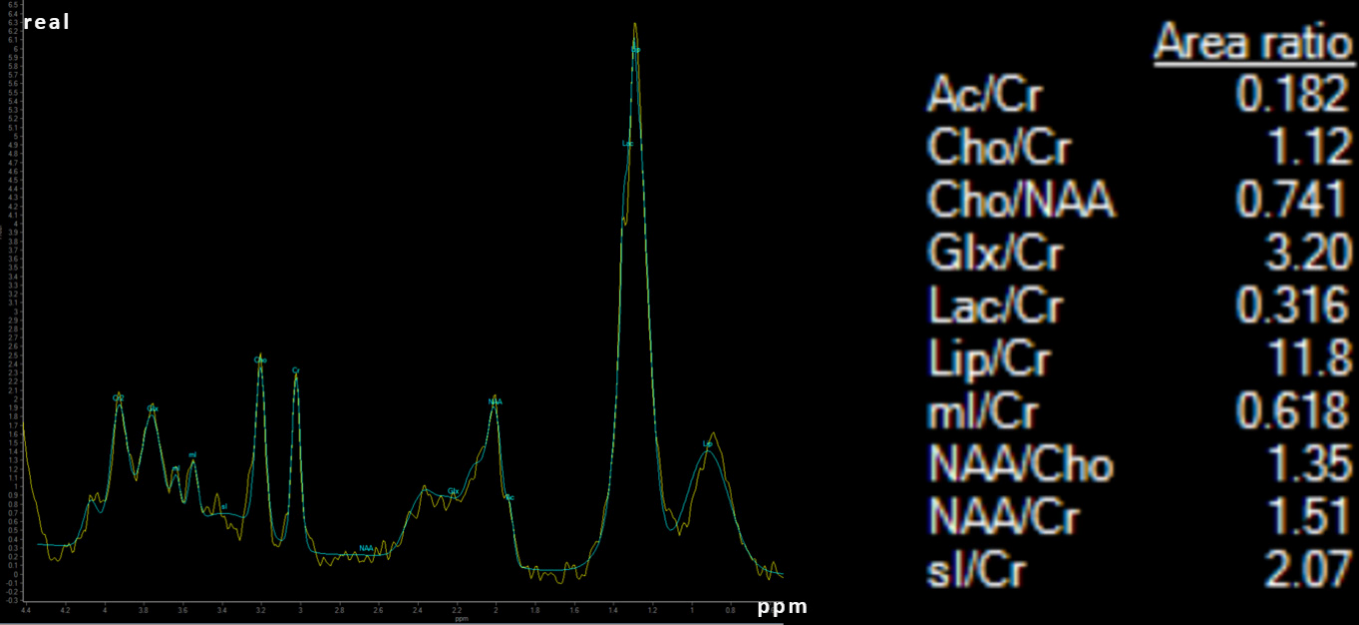
Single voxel spectroscopy with a voxel of 16 mm^3^ centered on the brain abscess.

Single voxel spectroscopy with a voxel size of 16 mm^2^ and an echo time (TE) of 30 ms centered on the two lesions presented with a very high lipid peak (Lip/Cr ratio of 11.8), with a small lactate peak (Lac/Cr 0.316) with a decreased NAA (NAA/Cr ratio 1.51) and with slightly increased Cholin (Cho/Cr ratio 1.12 and Cho/NAA ratio 0.741) (Figure 3). Myoinositol and acetate were not elevated.

Morphological characteristics on DWI, SWI, *T*_2_W and post-contrast *T*_1_W images together with decreased rCBV in the contrast enhancing rim on DSC PWI and high FA value within the central necrosis on DTI ascertained the diagnosis of pyogenic brain abscess.

**Figure 4.**
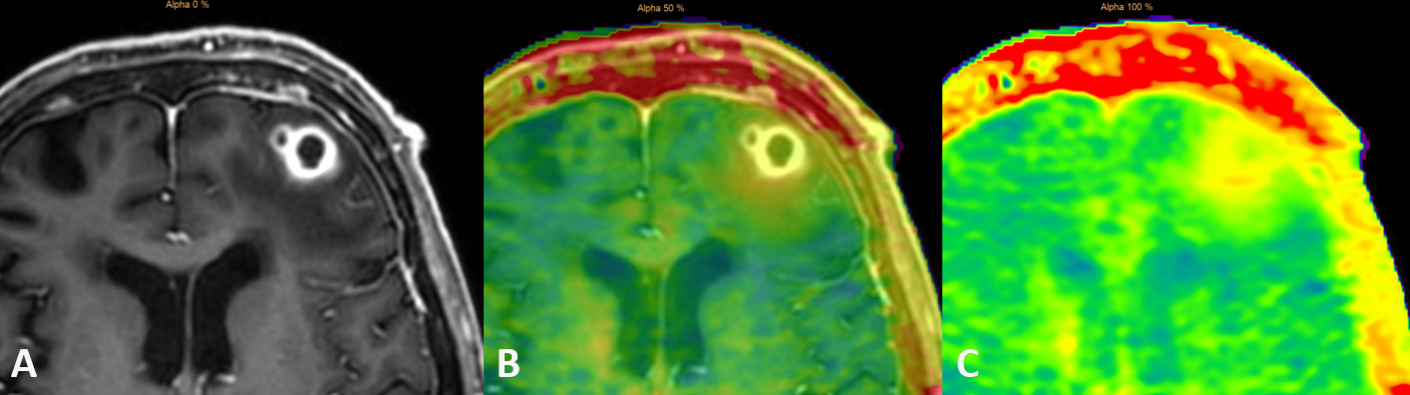
Postcontrast *T*_1_W TFE image (Figure 4A) underlaid on the APTw image (Figure 4B) and pure APTw image (Figure 4C). APTw,amide proton transfer weighted; *T*_1_W, *T*_1_weighted; TFE, turbo field echo.

**Figure 5.**
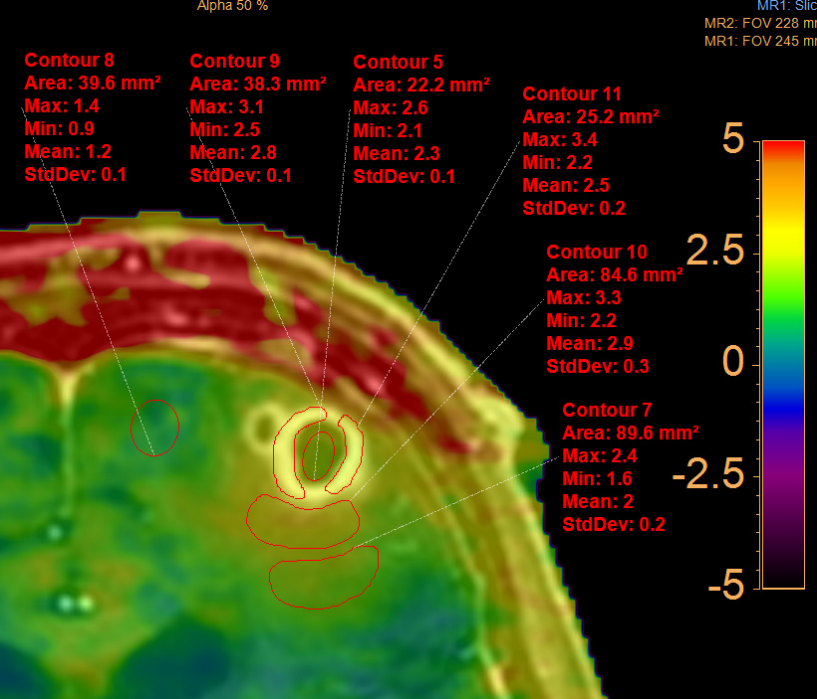
APTw values within the central necrosis, the contrast enhancing rim, the direct perifocal edema with inflammatory tissue (cerebritis) and the distant surrounding edema compared to APTw values of normal frontal white matter. APTw, amide proton transfer weighted.

**Figure 6.**
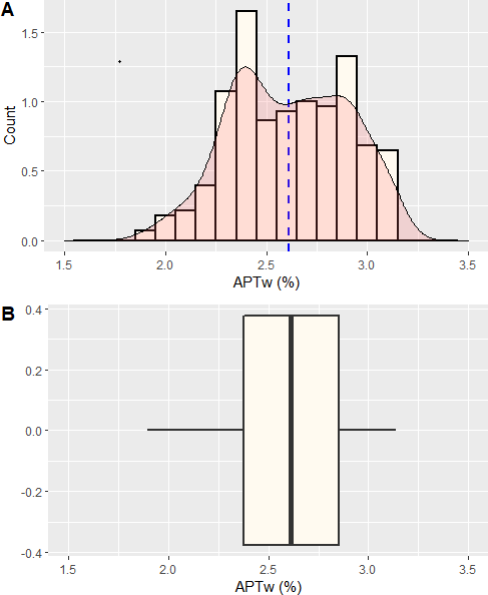
Histogram of the contrast enhancing rim. The histogram was computed with the statistical programming language “R v3.6.1” in the “R Studio” environment in combination with the packages “ggplot2”” and “cowplot”. Figure 6A shows the histogram with an overlaid density plot in red. The mean is depicted as a dashed blue line. It presents a double peak with APT values at 2.4% and 2.9% Figure 6B shows a boxplot of the data with the median as a prominent black line in the middle and the 25th and 75th percentile as the outer borders of the box. APT, amide proton transfer.

APTw images were underlaid, fused and coregistered with a *T*_1_W TFE post-contrast image on a separate workstation (Intellispace Portal^®^ Philips version 8) (Figure 4). This procedure allowed to switch from the *T*_1_W post-contrast TFE image (Figure 4A) to the mixed *T*_1_W / APTw image (each 50%, on Figure 4B) to the pure APTw image (Figure 4C). Free hand regions of interest (ROIs) were drawn on the *T*_1_W post-contrast TFE sequence and APTw values were obtained on the fused APTw image. The ROIs were drawn within the necrotic center, in the contrast enhancing rim, in the surrounding edema directly adjacent to the contrast enhancing rim, in the surrounding edema at approximately 1 cm distance to the contrast enhancing rim and in the normal frontal white matter (Figure 5). To assess the APTw values of the whole contrast enhancing rim, a voxelwise segmentation was performed on APTw images fused with postcontrast TFE images on a separate workstation (Intellispace Discovery ^®^Philips Healthcare, Best, the Netherlands). Based on the segmentation data, a histogram was then computed with the statistical programming language “R v3.6.1” in the “R Studio” environment in combination with the packages “ggplot2”” and “cowplot” (Figure 6).

The necrotic center had APTw values of 2.6%/2.3%/2.1% (maximum/mean/minimum), the directly surrounding edema had values of 3.3%/2.9%/2.2% , the more distant edema had values of 2.4%/2.0%/1.6% and the normal white matter had values of 1.4 %/1.2 %/0.9% (Figure 5). From the histogram of APTw values of the entire contrast enhancing rim (Figure 6), the following precise parameters were derived: minimum = 1.9%, 25th percentile = 2.4%, median = 2.6%, mean = 2.6%, 75th percentile = 2.9%, maximum = 3.1%, thus corresponding approximately to the APTw values of the contrast enhancing rim, obtained by manual ROI measurements (Figure 5) (with APTw 3.1–3.3% / 2.5–2.8% / 2.2–2.5%). Additionally, two distinct peaks within the contrast enhancing rim were observed at 2.4 and 2.9% (Figure 6).

Following MR imaging, neurosurgical abscess aspiration of pus was performed and empirical treatment with Ceftriaxone, Metronidazole, Dexamethasone was started. Microscopy of the abscess showed filamentous Gram-positive rods. Nocardia Farcinica was identified on cultures by 16S-rRNA sequencing. Treatment was adjusted to Imipenem and Trimethoprim- Sulfomethoxazol (TMP-SXT). Additional work-up excluded overt immunosuppression and additional infectious foci, especially in the lungs. Due to severe side-effects of antibiotic therapy with renal failure, nausea, weight loss, TMP- SXT had to be discontinued and Meropenem was started. The patient's condition deteriorated and he died of respiratory failure on the 11th day after hospital admission.

The patient gave general written informed consent after admission to the hospital.

## Discussion

In this report, we describe the MR imaging findings of a cerebral Nocardiosis abscess in an immunocompetent patient and specifically provide previously unpublished APTw imaging characteristics. Lastly, we discuss the imaging findings in respect to possible differential diagnoses such as high grade tumorous brain lesions (*i.e.* high grade glioma and cerebral metastasis).

Nocardiosis is a localized or disseminated infection caused by Nocardia, an aerobic antinomycete with multiple species.^8–10^ The majority of Nocardia infections occur in patients with cell-mediated immunosuppressive conditions; however, up to 50% of patients with nocardial infections are immunocompetent.^3,10^ Nocardia brain infection can occur as an isolated^10^ or as a disseminated disease in two or more organs, especially with involvement of lungs, skin and brain.^3,8^ CNS infection occurs in up to 44% of patients with Nocardiosis,^3^ mostly presenting as meningitis and very rarely as brain abscess.^1,2^ Our patient was diagnosed with brain abscess due to Nocardia Farcinica. This species has a higher risk for dissemination to the brain than other Nocardial species,^8^ but brain abscesses are rare, often multifocal, poorly encapsulated, mostly located in frontal and parietal lobes^2,3,8^ and present with a very high mortality rate between 30 and 60%^2,3,8^ because the germ is often resistant to multiple antibacterial agents.^10^ A combined immediate approach of medical and surgical therapy is recommended.^3,8,10^

On MRI, the diagnosis of a pyogenic brain abscess could be established based on morphological MR imaging with central diffusion restriction on DWI, T1 hypointense rim, double rim sign on SWI, peripheral rim enhancement on *T*_1_W TFE images.^11^

Multimodality DSC PWI and DTI imaging confirmed the diagnosis of brain abscess with hypoperfusion/low rCBV of the contrast enhancing rim compared to normal white matter^12,13^ and with high FA values within the central necrosis.^14^ Nonetheless, certain imaging findings pointed towards a malignant brain tumor. Specifically, spectroscopy presented with a very high lipid peak and elevated cholin peak corresponding to a spectrum of a malignant tumor^13,15,16^ ; peaks specific for brain abscess as succinate at 2.4 ppm, acetate at 1.9 ppm, alanine at 1.5 ppm, aminoacids at 0.9 ppm and lactate at 1.3 ppm were absent.^15–17^ The perfusion parameters obtained by DCE perfusion (especially k-trans values and perfusion time curves) were also mimicking the appearance of tumorous lesions.^18–20^

Thus, to summarize, the imaging results from routine multimodality and multiparametric imaging (DTI, DSC, DCE perfusion and spectroscopy) were not fully conclusive.^4–6^ However it is already well known that differentiation between brain abscess and high grade glioma can be very difficult because imaging characteristics from multiparametric and multimodality imaging can be inconclusive.^4–6^ Thus, further imaging modalities aiding in ascertaining the correct diagnosis are of clinical interest.

We acquired APTw images of the patients' brain. APTw is a molecular imaging technique^7^ that has shown promise in differentiating infective mass lesions from tumorous lesions.^7,21^ In the future, APTw imaging may become a valuable asset in imaging infective mass lesions and imaging characteristics as described in this report may become of interest.

First, APTw values (maximum, mean, minimum) were higher in the direct perifocal edema surrounding the contrast enhancing rim of the Nocardia abscess compared to the APTw values of the contrast enhancing rim itself. This may reflect the fact that perifocal cerebritis surrounding the real abscess area is present as can be observed by faint contrast enhancement surrounding the abscess rim (Figure 1G–1H).

Second, the necrotic central part of the brain abscess had lower APTw values compared to the contrast enhancing parts but higher values compared to normal appearing white matter. These imaging characteristics differ from those of tumorous lesions as reported in previous studies, where the mean APTw value was described to be significantly lower in the necrotic region of high grade tumors or radiation necrosis compared to healthy contralateral regions.^22,23^ It can be suspected that in infectious necrotic areas the presence of inflammatory cells leads to increased protein and peptide content,^24–26^ and thus to increased APTw values compared to necrotic tumorous areas.

As for the contrast enhancing rim, the values in our patient were in the same range as those reported for metastatic and primary high grade brain tumors.^26^

Lastly, we observed two slight peaks in the histogram of the contrast enhancing rim of the brain abscess at APTw values of 2.4 and 2.9%. As these peaks have not been described for tumorous lesions, we speculate that they may potentially be specific for infective brain abscesses or even abscesses caused by Nocardia.^23–28^

In conclusion, we suspect that APTw imaging may provide additional insights in complex cases where multimodality and multiparametric MR imaging with DTI, PWI and spectroscopy is inconclusive. However, it has to be strongly emphasized, that the imaging characteristics described in the present work are only based on the imaging study of a single patient. Thus, future studies based on larger patient series should evaluate the utility and added value of APTw imaging in the imaging of infective mass lesions.

## Learning points

1. The diagnosis of a cerebral nocardiosis abscess may be very challenging, even when images from multimodality MR imaging are available.
2. Cerebral nocardiosis abscesses may mimic the appearance of tumorous lesions on images obtained from multimodality MR imaging.
3. APTw imaging showed distinct imaging characteristics of the brain abscess. APTw may possibly be a useful tool to differentiate tumorous lesions from pyogenic brain abscesses.
